# A cross-sectional analysis of the relationship between ischemic stroke and uric acid in young people in highland areas

**DOI:** 10.3389/fendo.2023.1325629

**Published:** 2024-01-11

**Authors:** Yifan Yang, Li Gao, Fuwei Shen, Jiaodan Miao, Hua Liu

**Affiliations:** Department of Neurology, the Affiliated Hospital of Southwest Jiaotong University & The Third People’s Hospital of Chengdu, Chengdu, Sichuan, China

**Keywords:** ischemic, young people, risk factor, SUA, highland

## Abstract

**Objective:**

To investigate the relationship between serum uric acid (SUA) levels and ischemic stroke in young patients in the Ganzi Tibetan plateau area.

**Methods:**

A cross-sectional survey was undertaken from January 2020 to June 2023 involving young patients (age: 15–45 years) diagnosed with ischemic stroke. The survey was conducted at the Department of Internal Medicine of the People’s Hospital of Derong County, Ganzi Prefecture. The participants underwent a comprehensive assessment, including questionnaire surveys, physical examinations, laboratory tests, and head computed tomography (CT) examinations. Based on the tertiles of serum uric acid (SUA) levels, the patients were stratified into three groups. Furthermore, stroke severity was classified into mild (1–4 points), moderate (5–15 points), and severe (>15 points) categories using the National Institute of Health Stroke Scale.

**Results:**

The severe stroke group exhibited higher levels of age, glucose, systolic blood pressure, serum triglyceride, low-density lipoprotein cholesterol, and serum uric acid (SUA) compared to the mild stroke group (P < 0.05). Furthermore, the likelihood of male sex, advanced age, smoking, and a family history of stroke, diabetes mellitus, and heart disease were significantly elevated in the severe stroke group compared to the moderate stroke group (P < 0.05). Multivariate logistic regression analysis conducted on young adults residing in highland areas revealed a significant association between SUA levels and the risk of stroke.

**Conclusion:**

Elevated SUA levels serve as a distinct risk factor for the development of a major stroke in young patients in highland areas. At SUA levels of 320.56 mol/L, the risk of a moderate-to-severe stroke is noticeably elevated.

## Introduction

Acute cerebrovascular accidents fall within the category of cerebral infarctions, wherein localized ischemia in the brain tissue is the main symptom. Stroke predominantly affects older age groups, yet the Global Burden of Disease Survey estimates that an additional 2 million young people worldwide experience strokes each year. A 2019 study discovered that stroke onset can occur up to 10 years earlier in China than in developed Western nations. As the world’s largest developing country, China has a young population with stroke typically classified internationally between the ages of 18 and 50 years. However, considering the average age of stroke onset in China is 60 years, it is reasonable to define the young stroke population as individuals under 45 years old (1, 2). Since the 1980s, there has been a rise in the prevalence of vascular risk factors and substance addiction among the younger population, coinciding with an increase in ischemic stroke incidence in young adults. Young people are particularly susceptible to various risk factors for stroke such as pregnancy, oral contraceptive use, inadequate physical exercise, excessive alcohol consumption, and smoking. Poor dietary habits also pose a significant risk factor for young adults compared with that for older patients. The frequency of strokes can be influenced by geographical factors, climate circumstances, ethnicity, and lifestyle. The Qinghai-Tibet Plateau, the third polar region globally, exhibits unique climatic characteristics, including low pressure, low temperature, and thin air, directly impacting human health and resulting in various illnesses. For instance, cold conditions can elevate blood viscosity, fibrin levels, and the time it takes for a blood clot to form, increasing the risk of stroke and causing arterial spasms and vascular embolism. Despite being the southernmost tip of the Tibetan Plateau, the birthplace of the Tibetan people, and a bordering region to the Tibet Autonomous Region, the Ganzi Tibetan region in Sichuan Province has experienced only modest economic growth. Derong County, situated in Ganzi Prefecture, Sichuan Province, lies between latitudes 28°09' and 29°10' North and longitudes 99°07' and 99°34' East. The county boasts a maximum elevation of 5599 meters and maintains an average elevation of 2700 meters. Derong County encompasses 2,916 square km, comprising 1 township, 11 townships, 127 villages, 2 communities, and 245 natural villages, with a population of approximately 26,000 people. Local Tibetans in Derong County exhibit higher average systolic blood pressure (SBP) and diastolic blood pressure (DBP) than Han Chinese, primarily due to excessive salt intake. Additionally, the high incidence of hypertension among Tibetans may be attributed to their residence in mountainous terrain, where long-term alpine hypoxia stimulates the vascular center, causing peripheral vasoconstriction, increased resistance, and reduced arterial elasticity. The Tibetan diet structure, dominated by tsampa, ghee, and other high-salt and high-fat foods, also contributes to the occurrence of metabolic syndromes such as hyperlipidemia, hyperglycemia, and hyperuricemia.

The final byproduct of purine nucleotide metabolism is the weak organic acid, SUA. Maintaining a balance between the production and excretion of SUA by the kidneys is crucial for managing SUA levels in the body. SUA can be produced either from exogenous sources with high purine intake, such as meat, fish, and animal organs, or from endogenous sources like tissue breakdown and the resynthesis of purines from RNA and DNA bases (3). Hyperuricemia exerts a pathophysiological impact on several diseases, encompassing gout, chronic renal disease, cardiovascular disease, and metabolic syndrome (involving obesity, hypertension, and hyperlipidemia) (4, 5). Several studies propose that excessively elevated SUA levels may also be linked to additional risk factors for cerebrovascular diseases, such as hypertension, diabetes, and hyperlipidemia. This association has the potential to disrupt the body’s redox state, hasten the development of atherosclerosis, and significantly contribute to the risk of stroke (6). Numerous studies (7–9) have demonstrated that hyperuricemia increases the risk of and mortality from stroke. Between January 2020 and June 2023, the author examined young patients with ischemic stroke admitted to the Internal Medicine Department of Derong County People’s Hospital, focusing on the potential for neighborhood public health services. The analysis presented below aims to help readers understand the relationship between local stroke and SUA.

## Subjects and methods

### Study subjects

From January 2020 to June 2023, a cross-sectional survey was conducted among young patients with ischemic stroke (age: 15–45 years) admitted to the Department of Internal Medicine of the People’s Hospital of Derong County, Ganzi Prefecture, who underwent questionnaire survey, physical examinations, laboratory tests, and head computed tomography (CT) examinations. The inclusion criteria were as follows: (1) Diagnosed with ischemic stroke according to the 2018 Chinese guidelines for the diagnosis and management of acute ischemic stroke; (2) Age between 15 and 45 years; (3) Onset of initial symptoms within 7 days; (4) No history of cerebrovascular disease; and (5) Verification by head CT imaging. The exclusion criteria were as follows: (1) Aged <15 or >45 years; (2) Transient ischemic attack in the brain; (3) Malignancy, hematologic diseases, or autoimmune diseases; (4) Use of drugs affecting SUA metabolism (e.g., diuretics, antigout drugs) within 3 months; (5) Other strokes of determined etiology. All study participants provided informed consent by signing the informed consent form. The study received approval from the Ethics Committee of Derong County People’s Hospital (ethics number: (2021)-T-39).

### Surveys and testing methods

Data were collected, and physical examinations were conducted on eligible patients by uniformly trained specialists. Each participant underwent a questionnaire survey, a medical history interview, a physical examination, a blood biochemical analysis, and a head CT imaging analysis.

### Questionnaire survey

The questionnaire covered general personal information; current medical history; history of atrial fibrillation or cardiac valve disease, hypertension, ischemic stroke, hyperlipidemia, or diabetes mellitus; and family history. General conditions such as age, sex, smoking, drinking, and medication history were included.

### Physical examination

An examination of the body height, body mass index (BMI), and blood pressure (BP) were measured as part of the physical examination.

### Laboratory test

Subjects fasted for more than 12 h before blood collection. Fasting elbow venous blood obtained in the morning was analyzed for fasting blood sugar, total cholesterol (TC), triglyceride, low-density lipoprotein (LDL)-C, high-density lipoprotein (HDL)-C, and SUA using a Myriad BS-860 automatic biochemical analyzer.

### Imaging examination

All patients underwent a head CT examination within 24 h after admission, using SOMATOM go.Fit 62 rows for head CT examination, with screening staff having more than 3 years of experience in CT examination.

### Assessment of the severity of neurological impairment

The severity of neurological deficits in the young stroke group was assessed according to the National Institute of Health Stroke Scale, categorizing strokes as mild (1–4 points), moderate (5–15 points), or severe (>15 points) (10).

### Statistical analysis

All data were statistically analyzed using SPSS 20.0 software. Measurement data were expressed as mean ± standard deviation (x ± s), and a t-test was used for comparison. Count data were expressed as a rate (%), and the chi-square test was employed. Logistic regression analysis was used for multiple factors, and a significance level of P < 0.05 was considered statistically significant.

## Results

### SUA levels and grouping

The population was categorized into three groups: (1) the first group: 70 (33.3%) with SUA levels of ≤320.56 μmol/L; (2) the second group: 70 (33.3%) with SUA levels of 320.56–424.36 μmol/L; and (3) the third group: 70 (33.3%) with SUA levels of ≥424.36 μmol/L.

### Demographic and clinical characteristics of the study population

Though no significant differences were observed in age, sex, history of smoking, history of alcohol consumption, history of heart disease, DBP, and HDL levels between the second and third groups compared to the first group, notable differences were found in the composition of history of diabetes, family history of stroke, history of SUA medication, SBP, BMI, and serum glucose, triglyceride, and TC levels as the SUA level increased (P < 0.05). The third group exhibited statistically significant differences (P < 0.05) with greater composition ratios for family history of stroke, history of diabetes mellitus, history of SUA treatment, BMI, and serum TG, glucose, and LDL-C levels than the second group (**Table 1**).

**Table 1:** Demographic and clinical characteristics of the study population.

Items	First quartile group	Second quartile group	Third quartile group	P
**SUA (μmol/ L**)	≤320.56	325.56~424. 36	≥424.36	
Number of people , n(cases)	70	70	70	
Age, mean (SD), years	32.10±6.17	33.42±5.68	34.58±5.52	0.921
Male, n(%)	38 (54.28%)	40(57.14%)	37(52.85%)	0.426
Smoking, n(%)	33 (47.14%)	36(51.42%)	35(50%)	0.488
Alcohol use, n(%)	54 (77.14%)	58(82.86%)	60 (85.71%)	0.632
Family history of stroke, n (%)	6 (8.5%)	18 (25.71%)*	24 (34.28%) *△	0
History of diabetes mellitus, n (%)	8 (11.42%)	12 (17.14%) *	28 (40%) *△	0
History of heart disease, n ( %)	2 (2.8%)	4 (5.7%)	4 (5.7%)	0.684
SBP, mmHg	131. 58 ± 12.20	140.59 ± 13.26 *	144.11 ± 16.02 *	0
DBP, mmHg	82.16 ± 10.62	82.25 ± 10.69	82.72 ± 11.57	0.352
TG( mmol / L)	1.32 ± 0.68	1.78 ± 0.95	2.32 ± 1.24*△	0.000
TC( mmol / L)	4.39 ± 0.82	4. 73 ± 0. 95	5.12 ± 0.78 *	0.006
HDL-C( mmol / L)	1.28 ± 0.48	1.31 ± 0.46	1.36± 0.26	0.848
LDL-C( mmol / L)	2.47 ± 0.68	2. 73 ± 0.76	2.95 ± 0.78*△	0.017
GLU( mmol / L)	5.18 ± 2.08	4.97 ± 0.96	6.78 ± 2.16*△	0.000
Taking uric acid-lowering drugs, n (%)	2(2.8%)	8(11.42%)*	22(31.42%)*△	0.000

Compared with the first quartile group, *P < 0. 05; compared with the second quartile group, △P < 0. 05.

SBP, systolic blood pressure; DBP, diastolic blood pressure; BMI, body mass index; TG, triglyceride; TC, total cholesterol; HDL-C, high density cholesterol; LDL-C, low density cholesterol; GLU, glucose, SUA, serum uric acid.

### Comparison of risk factors in patients with different severity of neurological deficits

**Figure 1:**
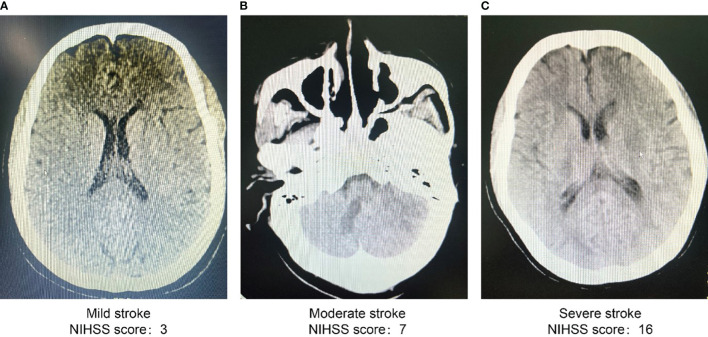
Head CT scan images of patients with different severity of stroke (Grouped by NIHSS scores). **(A)** Images of head CT scan of patients with mild stroke (NIHSS score 3). **(B)** Images of head CT scan of patients with moderate stroke (NIHSS score 7). **(C)** Images of head CT scan of patients with severe stroke (NIHSS score 16). CT, computed tomography scan; NIHSS, national institutes of health stroke scale.

The study participants included 33 patients with severe stroke with National Institute of Health Stroke Scale scores of at least 15, distributed as 9 in the first group, 12 in the second group, and 15 in the third. In the severe stroke group, age; male sex; smoking history; family history of stroke, smoking, diabetes mellitus, and heart disease; and serum glucose, SBP, TG, LDL-C, and SUA levels were all higher than those in the mild stroke group (P < 0.05). Additionally, the severe stroke group exhibited higher serum glucose and SUA levels than the moderate stroke group (P < 0.05). Composition ratios of men, family history of stroke, smoking, diabetes mellitus, age, and serum glucose and SUA levels were higher in the severe stroke group than in the other two groups (P < 0.05) (**Table 2**). We also selected head CT imaging of patients with different severity of stroke (**Figures 1A–C**).

**Table 2:** Comparison of risk factors in patients with different severity of neurological deficits.

Items NIHSS score	Mild stroke (1~4)	Moderate Stroke (5~15)	Severe Stroke (>15)	P Value
Number of people,n (cases)	92	85	33	0.000
Age, mean (SD), years	30.10±5.17	35.42±5.68*	38.58±7.22*△	
Male, n(%)	49(53.26%)	56(65.88%)*	24(72.72%)*△	0.000
Smoking history, n(%)	33(35.87%)	68(83.52%)*	30(90.90%)*△	0.000
Family history of stroke, n(%)	8(8.69%)	18(21.18%)*	14(42.42%) *△	0.000
History of diabetes mellitus, n (%)	10(10.87%)	36(42.35%) *	14(42.42%) *△	0.000
History of heart disease, n ( %)	4(4.3%)	6 (7.05%)	5(15.15%)*△	0.034
SBP, mmHg	135.48 ± 15.48	139.55 ± 13.52	145.21 ± 16.16*	0.000
DBP, mmHg	80.26 ± 9.62	82.25 ± 10.69	82.72 ± 11.57	0.053
BMI( kg / m2)	24.98 ± 4.98	25.59 ± 3. 58	25.80 ± 4.18	0.589
TG( mmol / L)	1.45 ± 0.88	1.77 ± 1.32*	1.81 ± 1.16*	0.026
TC( mmol / L)	4.48 ± 2.16	4.84 ± 1.07	4.98 ± 1.17	0.384
HDL-C( mmol / L)	1.35 ± 0.43	1.31 ± 0. 89	1.28 ± 0.45	0.215
LDL-C( mmol / L)	2.44 ± 0.86	2.83 ± 0.88*	2.94 ± 0.92*	0.000
GLU( mmol/L)	5.44 ± 1.34	6.53 ± 3.64*	6.87 ± 1.86*△	0.000
UA(μmol/L)	319.68±123.67	349.89±103.07*	394.12±124.83*△	0.000
SUA level [n ( %)]				
First quartile group	36( 39.13%)	28( 32.94%)*	6(18.18%)*△	0.000
Second quartile group	29( 31.52%)	29(34.11%)*	12(36.36%) *△	0.000
Third quartile group	27(29.03%)	28( 32.94%)*	15( 45.45%) *△	0.000

Compared with the first quartile group, *P < 0. 05; compared with the second quartile group, △P < 0. 05.

SBP, systolic blood pressure; DBP, diastolic blood pressure; BMI, body mass index; TG, triglyceride; TC, total cholesterol; HDL-C, high density.

### Correlation between SUA levels and stroke occurrence

The quantitative link between variables and their interactions can be further elucidated through regression analysis. In this study, the stepwise regression method is employed to systematically introduce independent factors, exclude independent variables that do not significantly impact the dependent variable, and formulate a regression equation model. This model is designed to affirm the direction and proximity of the relationship between variables. The partial regression coefficient is denoted by “B” in the table, while the significance level is indicated by the “P” value. Moreover, insights into the influence of independent variables on the dependent variable can be gleaned from the regression coefficient “B” value. When the significance “P” of the independent variable is less than 0.05, it passes the significance test of the model at the 5% significance level and possesses valid statistics. Notably, a positive “B” value signifies a positive influence, whereas a negative value signifies a negative influence. In conclusion, **Table 3** reveals a significant positive relationship between age and smoking history, diabetes history, LDL-C (mmol/L), glucose (mmol/L), and SUA levels, with a significance of less than 0.05 and a positive regression coefficient. This suggests that SUA levels in patients in the highland region, our main study focus, significantly impact stroke in young people.

**Table 3:** Logistic regression analysis of risk factors and the effect of stroke.

Items	B	SE	Wald	df	P	Exp(B)	95% CI	
							Lower limit	Upper limit
Age	0.196	0.044	19.906	1	0.000	1.217	1.116	1.326
Smoking history	2.195	0.480	20.900	1	0.000	8.976	3.503	22.998
History of diabetes mellitus	2.106	0.578	13.261	1	0.000	8.216	2.645	25.522
LDL-C( mmol / L)	0.643	0.265	5.894	1	0.015	1.903	1.132	3.199
GLU( mmol / L)	0.531	0.112	22.371	1	0.000	1.701	1.365	2.119
SUA(μmol/ L)	0.616	0.284	4.714	1	0.030	1.852	1.062	3.230
Constants	-14.342	2.289	39.245	1	0.000	0.000		

LDL-C, low density cholesterol; GLU, glucose; SUA, serum uric acid; CI, confidence interval.

Variables with a significance greater than 0.05 were deemed to have a weak effect on the dependent variable and were excluded from the model, demonstrating no significant impact.

### Correlation between SUA levels and stroke severity

The significance analysis of age, smoking history, family history of stroke, and SUA and glucose (mmol/L) levels in **Table 4** indicates a significance level of less than 0.05, with a positive regression coefficient. This suggests a significant positive effect on the severity of stroke. Therefore, among young individuals in highland areas, there is a strong relationship between SUA levels and the severity of stroke. The dependent variable has been removed from the model and is not considered significant. However, the significance of the other variables was greater than 0.05, indicating a modest effect on it.

**Table 4:** Logistic regression analysis of risk factors on stroke severity.

Items	Unstandardized coefficient		Standardization factor	t	P
	B	SE	Beta		
Constants	-0.216	0.220		-0.981	0.328
History of diabetes mellitu	0.501	0.083	0.331	6.073	0.000
Age	0.030	0.006	0.256	4.921	0.000
Smoking history	0.373	0.080	0.251	4.660	0.000
Family history of stroke	0.276	0.093	0.151	2.959	0.003
Serum uric acid, SUA(μmol/ L)	0.129	0.044	0.146	2.911	0.004
Glucose, GLU( mmol / L)	0.032	0.013	0.130	2.479	0.014

LDL-C, low density cholesterol; GLU, glucose; SUA, serum uric acid.

## Discussion

SUA, identified as a pro-inflammatory substance, has been associated with a heightened risk of ischemic stroke in several studies (11). The proposed direct involvement of hyperuricemia in the clinical onset of cerebrovascular disease is linked to the association between elevated SUA and increased oxygen-free radical production (12, 13). During ischemia, the production of reactive oxygen species, particularly superoxide anions, escalates as tissue antioxidants decline, leading to a lower concentration of antioxidants in the tissue (14). Elevated SUA levels have been correlated with increased levels of systemic inflammatory mediators, vascular smooth muscle proliferation, and heightened platelet adhesion, all contributing factors to thrombosis (15, 16). Consequently, hyperuricemia may play a pathogenic role not only in the occurrence of ischemic stroke but also in the development of vascular stiffness (17, 18), atherosclerosis, and hypertension (19, 20). Moreover, SUA impedes the activation of nitric oxide synthase and nitric oxide production by insulin and vascular endothelial growth factor, leading to increased levels of reactive oxygen species and eventual harm to the vascular endothelium (21). Nevertheless, these factors may be associated with endothelial dysfunction, heightened levels of circulating systemic inflammatory mediators, thrombosis, and an escalation in oxidative stress (22). The specific pathogenic mechanisms of SUA in cerebrovascular disease, however, remain largely unknown.

After an ischemic stroke, the SUA levels tend to rise due to its capability to scavenge free radicals (23). According to Ji Man Hong (24), SUA levels initially decrease after the onset of stroke symptoms before gradually increasing over seven days. Numerous investigations in various countries have identified SUA as a favorable predictor of acute ischemic stroke (25). In the highland Ganzi Tibetan Autonomous Prefecture, the diet is rich in salt and fat, with staples like tsampa (barley fried and ground into noodles) and ghee (a product made from yak milk). Additionally, the locals have a high alcohol consumption, with an average exceeding 61 g/day. Harmful drinkers (alcohol consumption >61 g/day) have a 1.6 times higher risk of SUA (26), attributed to factors such as purine compounds in alcohol, lactic acid buildup affecting SUA excretion, encouragement of adenine nucleotide conversion raising SUA production, liver damage affecting purine conversion, and alcohol consumption often accompanying foods high in purines (27). Locals are less inclined to undergo regular medical checkups due to lower health awareness and a more traditional mindset. This predisposes them to atherosclerosis, contributing to metabolic syndrome encompassing hypertension, hyperlipidemia, diabetes, and hyperuricemia. Studies have reported that cardiovascular disease is arguably the disease for which there is the most evidence of a social gradient in incidence and risk. Low socioeconomic status is associated with a 55% increased risk of death from ischemic heart disease among adults aged 30-59 years in men (relative risk [RR] 1.55, 95% CI 1.51-1.60) (28). The disadvantage is that the educational level of the patients was not collected in this research study for the time being. The study indicates a significant difference (P < 0.05) in SUA levels between young patients with moderate-to-severe stroke and those with mild stroke. This suggests a potential influence of elevated SUA levels on neurological disabilities in patients with stroke, possibly linked to SUA-induced oxidative stress. The investigation identified a correlation between rising SUA levels and increased proportions of men with a history of diabetes, stroke, smoking, or alcohol use, alongside aging BMI, SBP, and fasting blood glucose, TG, and LDL levels. Multivariate logistic regression analysis recognized SUA levels as an independent risk factor for stroke severity, emphasizing their significant impact on stroke in young individuals. The study hypothesizes that SUA levels heighten the risk of severe stroke in individuals already predisposed to a high risk of stroke. This suggests that SUA levels could serve as a potential free risk factor for the progression of serious stroke, highlighting the need for further exploration into this relationship. The investigation, centered on the relationship between SUA levels and stroke severity, establishes a significant link among young individuals residing in highland areas. This finding underscores the importance of understanding and addressing SUA levels as a potential modifiable risk factor in stroke prevention strategies for this population.

There has been considerable discussion about the impact of hyperuricemia on patients with cerebral dead tissue. Extensive research has demonstrated that SUA functions as a mediator between oxidative pressure and endothelial function. Sudden changes in the SUA levels may disrupt the physiological components of vital tissues, leading to apoplexy, worsening of hypertension, and eventually resulting in ischemic cerebrovascular disease. Moreover, a significant increase in uric acid may indicate increased xanthine oxidase activity (29), which is known to produce superoxide and play a crucial role in pathogenesis (30). Expanded xanthine oxidase activity, a substantial source of reactive oxygen species, has been suggested to contribute significantly to organ damage in animal models of hypertension (31, 32). Meta-studies by Kim et al. in 2009 and Li et al. in 2013 supported the direct link between elevated SUA levels and stroke (22, 33). Results from a meta-analysis of 16 prospective studies, including 238,449 adults and adjusting for multivariate risk factors, suggested that hyperuricemia was associated with a significantly increased risk of stroke incidence and mortality (+47% and +26%, respectively). However, research has also indicated the potent cancer-prevention and cancer-mitigation effects of SUA (34). Serum concentrations of uric acid are almost 10 times higher than concentrations of other antioxidants in the body (35, 36). SUA has been suggested to play a role in neuroprotection following a stroke (37). High uric acid levels at onset are a biomarker of better prognosis in patients with acute ischemic stroke. Insufficiently low SUA levels may fail to confer neuroprotection against injury.

The role of hyperuric acid in individuals with acute cerebral infarction remains intricate, and extant research yields divergent outcomes, indicating both conceivable advantageous and detrimental ramifications of elevated uric acid levels (14, 16, 36, 38, 39). We posit that during the acute phase of cerebral infarction, the neuroprotective function of uric acid, attributed to its capacity as a free radical scavenger and bestowing antioxidant shielding, serves to mitigate oxidative stress and inflammation (40). This mitigation is associated with a diminution in neuronal damage and an enhancement in the prognosis of cerebral ischemia. Consequently, we propose a temporary abstention from SUA-decreasing therapy during this acute phase. Conversely, heightened SUA levels in the acute phase of non-cerebral infarction may induce oxidative stress and inflammation due to its pro-oxidant effects, rendering it a high-risk factor during cerebral infarction. At this critical juncture, patients are advised to maintain SUA at a relatively stable level (3-5 mg/dL) (38, 41). Consequently, SUA-controlling interventions should be tailored based on the specificities of the cerebral infarction. Precision in treatment recommendations can be achieved by diligently evaluating potential advantages and risks, while considering the patient’s overall health, comorbidities, and the distinctive characteristics of the infarction.

On the other hand, the current examination encounters specific challenges. Firstly, it adopts a cross-sectional observational approach devoid of a comprehensive follow-up plan. The study is confined to the population of Derong District in Ganzi Prefecture, thereby limiting the generalizability of the conclusions. Secondly, SUA levels were solely assessed at the point of confirmation, neglecting potential temporal fluctuations (42). Future investigations, incorporating repeated measurements and obtaining patient consent, are imperative to accurately capture changes in SUA levels and their correlation with stroke outcomes. Moreover, the intricate analysis of the role of serum uric acid, subject to variations across different stages of stroke, is impeded by the absence of pertinent information in the studies encompassed. In addition, there is a potential association between socioeconomic status, educational attainment, elevated SUA, and the incidence of cardiovascular disease (CVD) (43). Regrettably, the current study lacks data regarding the educational background of the participants, precluding an exploration of potential associations between education and the incidence of stroke. Overall, the potential influence of commonly prescribed medications on SUA levels is not treated as a confounding factor, warranting consideration in forthcoming research endeavors. Expanding the sample size and conducting follow-up studies in diverse geographical settings will enrich our comprehension of the intricate relationship between distinct SUA levels and the severity of stroke.

## Conclusion

In conclusion, elevated SUA levels emerge as a significant risk factor for the severity of stroke recovery. At SUA levels of >320.56 μmol/L, there is a notable increase in the risk of a moderate-to-severe stroke. This finding suggests that SUA may contribute to the severity of an enlarged stroke through certain pathophysiological processes.

## Data availability statement

The original contributions presented in the study are included in the article/supplementary material. Further inquiries can be directed to the corresponding authors.

## Ethics statement

Derong County People’s Hospital’s ethics committee granted approval for the study (ethics number: (2021)-T-39). The studies were conducted in accordance with the local legislation and institutional requirements. The participants provided their written informed consent to participate in this study. Written informed consent was obtained from the individual(s) for the publication of any potentially identifiable images or data included in this article.

## Author contributions

YY: Project administration, Writing – original draft, Writing – review & editing. LG: Funding acquisition, Methodology, Supervision, Writing – review & editing. JM: Writing – review & editing. FS: Writing – review & editing. HL: Conceptualization, Resources, Software, Writing – review & editing.
